# An Update on the Role of MRI in Treatment Stratification of Patients with Cervical Cancer

**DOI:** 10.3390/cancers15205105

**Published:** 2023-10-23

**Authors:** Amreen Shakur, Janice Yu Ji Lee, Sue Freeman

**Affiliations:** Cambridge University Hospitals NHS Foundation Trust, Cambridge CB2 0QQ, UK; amreen.shakur@nhs.net (A.S.); janice.lee1@nhs.net (J.Y.J.L.)

**Keywords:** gynaecological malignancy, cervical malignancy, FIGO staging, MRI

## Abstract

**Simple Summary:**

Magnetic resonance imaging (MRI) has a pivotal role in accurately staging cervical cancer and has been formally incorporated into the 2018 FIGO staging system. MRI can accurately assess tumour size and local and distant invasion as well as lymph node involvement, which is essential for triaging patients into surgical or chemotherapeutic management. In this review, we highlight key MRI findings and pitfalls pertaining to the updated FIGO stages and their implications for treatment selection into surgery or chemoradiation.

**Abstract:**

Cervical cancer is the fourth most common cancer in women worldwide and the most common gynaecological malignancy. The FIGO staging system is the most commonly utilised classification system for cervical cancer worldwide. Prior to the most recent update in the FIGO staging in 2018, the staging was dependent upon clinical assessment alone. Concordance between the surgical and clinical FIGO staging decreases rapidly as the tumour becomes more advanced. MRI now plays a central role in patients diagnosed with cervical cancer and enables accurate staging, which is essential to determining the most appropriate treatment. MRI is the best imaging option for the assessment of tumour size, location, and parametrial and sidewall invasion. Notably, the presence of parametrial invasion precludes surgical options, and the patient will be triaged to chemoradiotherapy. As imaging is intrinsic to the new 2018 FIGO staging system, nodal metastases have been included within the classification as stage IIIC disease. The presence of lymph node metastases within the pelvis or abdomen is associated with a poorer prognosis, which previously could not be included in the staging classification as these could not be reliably detected on clinical examination. MRI findings corresponding to the 2018 revised FIGO staging of cervical cancers and their impact on treatment selection will be described.

## 1. Introduction

Cervical cancer is the fourth most common gynaecological cancer worldwide, with a peak incidence between 25 and 40 years [1]. GLOBOCAN 2020 estimated that, worldwide, there were approximately 604 000 new cases of cervical cancer and 342 000 deaths due to the disease annually. Most new cases (approximately 90%) occur in low- and middle-income countries, where cervical cancer represents the third most common cancer in women.

One of the main risk factors is long-term or persistent infection with human papillomavirus (HPV). Over 70% of newly diagnosed cervical cancers are caused by either the HPV 16 or 18 subtypes. A further 19% of cervical cancers are caused by the HPV types 31, 33, 45, 52, or 58 [2]. HPV is a ubiquitous sexually transmitted infection with a prevalence of 11.7% globally, with a geographic distribution ranging from 2% to 42% [3]. The majority of HPV infections are cleared by women in two years, and only 10% cause a persistent infection.

This knowledge of HPV epidemiology has led the World Health Organisation (WHO) to call for a worldwide HPV eradication program [4]. The WHO global strategy proposes that a 90–70–90 target be met by 2030 for countries to be on the path towards eliminating cervical cancer. This target aims for 90% of girls to be fully vaccinated with the HPV vaccine by 15 years old, 70% of women to be screened with a high-performance test by 35 years of age and again by 45 years of age, and 90% of women affected by a cervical disease (precancer and invasive cancer) to receive treatment [4].

It has been postulated that the median cervical cancer incidence rate will fall by 42% by 2045 and by 97% by 2120 if these 90–70–90 targets are met [4].

Primary prevention through HPV vaccination of adolescent girls has been shown to be the most effective long-term intervention for reducing the risk of cervical cancer. Current guidelines to confer full protection are for two doses to be administered between the ages of 9 and 14 years. In addition to protecting against cervical lesions and cancer, they also reduce the risk of disease in the vulva, vagina, and anus. One study involving 60 million individuals with a follow-up period of 8 years after vaccination found that the prevalence of the various strains of HPV, anogenital warts, and high-grade cervical abnormalities (cervical intraepithelial neoplasia 2 and 3 (CIN2 and CIN3)) all significantly declined in all studied age groups. A separate study also found a significantly reduced risk of HPV-based invasive cervical cancer in the vaccinated population [3].

The Papanicolaou smear test (Pap smear) is a population-based cytological screening test that was effective in reducing the number of cervical cancers. However, the Pap smear required high levels of resources and suffered from variable quality assurance. HPV-based testing has replaced the Pap smear in many countries, including the UK, as it has improved sensitivity, accuracy, and reproducibility. HPV detection has increased the colposcopy referral rate and subsequently improved the detection rate of CIN3 and cervical cancers.

The transformation zone is the junction between the squamous epithelium of the ectocervix and columnar epithelium of the endocervical canal. Metaplasia occurs at the transformation zone, where columnar epithelium is replaced by squamous epithelium, and is the commonest site for cervical intra-epithelial neoplasia (CIN), which can progress to cervical cancer. The transformation zone is easily accessible for assessment by colposcopy using acetic acid. Areas of CIN or cervical cancer are revealed as acetowhite lesions. Under local anaesthetic, the lesion can be biopsied or a large loop excision of the transformation zone (LLETZ) performed. 

Cervical cancers are differentiated into different histological types, with the commonest being squamous cell carcinomas, constituting approximately 70-80% of cervical cancers. The glandular histological subtypes include adenocarcinomas, which account for a further approximately 25% of cervical cancers and are typically associated with a poorer prognosis [5]. Rarer subtypes include carcinosarcoma, adenosquamous carcinoma, and adenosarcoma.

## 2. The Role of Different Imaging Modalities in the Assessment of Cervical Cancer

Transvaginal ultrasound (TVUS) is considered the first-line imaging investigation for patients with gynaecological symptoms and is often more cost-effective and readily available than other imaging modalities, particularly in low-income countries. However, it is not part of routine cervical cancer detection and staging. Recent meta-analyses have shown TVUS to demonstrate comparable sensitivity and specificity for the estimation of tumour volume and presence of parametrial invasion; however, the technique is largely dependent upon operator skill and expertise. TVUS has a limited role in the evaluation of lymph node status, precluding it from becoming the primary imaging modality for cervical cancer assessment. However, TVUS in conjunction with transabdominal ultrasound (which may depict para-aortic lymph nodes or hydronephrosis) may have a role in the assessment of cervical cancer in resource-constrained areas where access to MRI is limited [6,7]. 

Computed tomography (CT) has fewer contra-indications, is quicker, and is usually more widely available when compared to MRI. The intrinsic lower soft tissue resolution of CT leads to reduced accuracy in the assessment of tumour size and parametrial invasion, although some studies have shown an accuracy of up to 86% in detecting cervical tumours on CT. Tsili et al. found that the relative hypo-enhancement of cervical tumours when compared to the background cervical tissue and the acquisition of thin sections with multiplanar reformats aid in tumour delineation. In comparison, MRI has up to 95% accuracy in the detection of cervical tumours. CT does have a role in the assessment of distant disease and can depict suspicious lymph nodes with a reported accuracy of 86% as well as identifying ureteric/pelvic sidewall involvement and distant metastases [8,9].

The role of F-fluorodeoxyglucose (FDG) positron emission tomography-computed tomography (PET/CT) in cervical cancer staging is well established due to its greater sensitivity in showing the presence of lymph node metastases and extra-pelvic disease extension when compared to CT [10]. Current guidelines recommend FDG-PET/CT in patients with stage IB1 disease or above who are eligible for surgical treatment and in patients with stage II–IVA disease to help assess for nodal and distant metastatic disease to guide therapeutic management [10,11,12]. 

The International Federation of Gynaecology and Obstetrics (FIGO) staging system is the most utilised classification system for cervical cancer worldwide, and the cervix was the first organ to be assigned a clinical staging system for cancer by FIGO in 1958. The most recent revision took place in 2018, previously having been revised in 2009, when staging was based on clinical evaluation alone [6]. The European Society of Urogenital Radiology (ESUR) recommended the inclusion of MRI into the staging classification in 2010 due to the high soft tissue resolution and accuracy in determining tumour size, parametrial invasion, pelvic sidewall invasion, and lymph node metastases [13]. MRI has also been shown to be cost effective as patients who underwent MRI as the initial imaging procedure for staging required fewer tests and procedures compared with those who underwent clinical staging alone [14]. The main changes in FIGO 2018 therefore relate to the utilisation of imaging to assign staging, which in turn led to the re-categorisation of stage IB into three size ranges and the inclusion of nodal disease as a new stage IIIC. 

### MRI Protocol for Uterine Cervical Cancer

Patient preparation is key to optimising imaging quality. A partially filled bladder ensures the uterus is in an optimal position, so patients are encouraged to void their bladder approximately half an hour prior to the examination so their bladder is partially filled during examination. Some centres encourage patients to fast for approximately 4–6 hrs prior to the study to reduce bowel peristalsis. An intramuscular injection of an anti-peristaltic agent (Buscopan) is administered before imaging is performed, which reduces motion artefact from bowel peristalsis. Whilst other methods including enemas and pelvic strapping are used for pelvic MRI for other indications, there is no substantial evidence to support their routine use for cervical cancer MRI [15].

**Figure 1:**
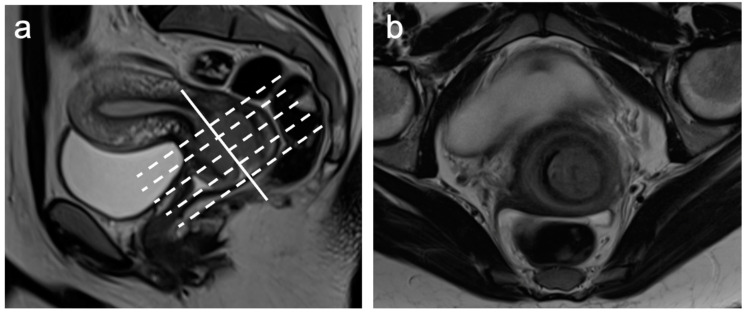
Sagittal T2WI (**a**) highlights the long axis of the cervix (solid white line) and the perpendicular axis to the cervix (dashed white lines) from which the (**b**) axial-oblique sequences are obtained.

A standard MRI protocol (Table 1) for cervical cancer staging involves obtaining a large field of view (FOV) sagittal T2-weighted image (T2WI); this is then used to plan higher-resolution small FOV imaging perpendicular to the long axis of the cervix, important for local staging of the tumour and accurate assessment of the parametrium (Figure 1). The normal zonal anatomy of the cervix is best depicted on T2WI with the central endocervical glands and mucosa demonstrating hyperintense signal intensity, surrounded by a hypointense fibrous stroma and an outer intermediate signal intensity loose stroma, extending to the parametrium [16]. Cervical tumours, when visible, are best depicted on T2WI, where they appear as intermediate signal intensity lesions, which are readily distinguishable from the hypointense cervical stroma. DWI can aid tumour detection when the lesion is isointense to the background cervix.

ESUR guidelines recommend a large FOV axial T1-weighted image (T1WI) and T2WI obtained from the renal hila to the pubic symphysis to assess extra pelvic diseases, such as lymph node enlargement, bone involvement, and hydronephrosis [17,18]. 

Diffusion-weighted imaging (DWI) is a functional imaging technique that is sensitive to the microscopic motion of water molecules. With derived apparent diffusion coefficient (ADC) maps, it can be used to evaluate the molecular function and micro-architecture of biological tissue [19,20]. Different tissues have characteristic diffusion properties, and in tissues that are highly cellular, the diffusion of water molecules is relatively more restricted. This manifests as a high signal on DWI images, with a corresponding low signal on ADC maps. Several studies have demonstrated the added value of DWI both in the detection of tumours on initial staging, particularly those that are small and isointense on T2WI, as well as in detecting cervical cancer recurrence [18,19,21]. 

DWI should be acquired in the sagittal and axial oblique planes, with corresponding T2WI for anatomical correlation [22]. For accurate analysis, images should be acquired with a minimum of two b values and must be corroborated with the corresponding ADC maps to avoid the potential pitfall of overcalling diffusion restriction in tissues that have an inherently high T2 signal (T2 shine-through phenomenon). The presence of post-biopsy oedema is a common cause of this pitfall [23]. Whilst malignant cells generally demonstrate restricted diffusion, benign entities including blood products, abscesses, and keratin may also exhibit restricted diffusion, highlighting the importance of correlation with T1WI and T2WI to avoid this pitfall [21].

DWI has also emerged as a potential biomarker for assessing treatment response to chemoradiotherapy in cervical cancer. A meta-analysis analysis performed by Harry et al. assessed the role of DWI and ADC in predicting treatment response [24]. They demonstrated a statistically significant correlation between ADC values detected within three weeks of treatment as well as the percentage change in ADC values during this period with overall treatment response. Therefore, a change in ADC values rather than absolute ADC values may serve as a suitable marker in the determination of early response. However, they did not demonstrate a significant relationship between pre-treatment ADC values and treatment response, and therefore cannot be used to determine initial treatment selection [24,25]. 

**Figure 2:**
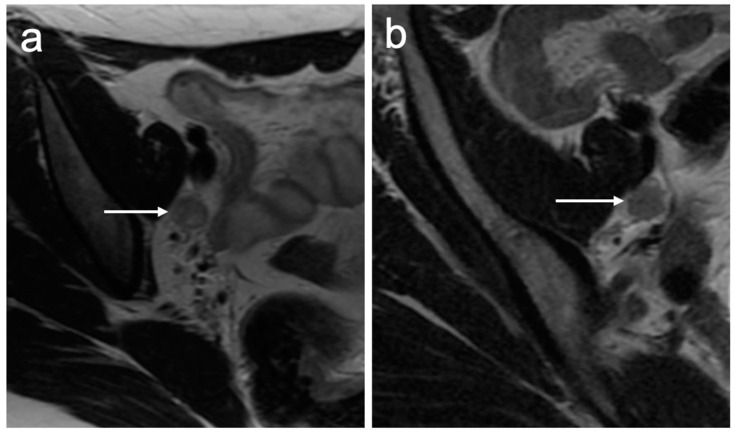
Axial T2WI images demonstrate typical appearances of metastatic lymph nodes with (**a**) a rounded morphology and central necrosis and (**b**) irregular spiculated margins.

DWI facilitates the detection of lymph nodes; however, it is important to note that both physiological and pathological nodes demonstrate diffusion restriction. T1 and T2 weighted imaging can be used to further evaluate the morphology of the lymph node. Suspicious features include a rounded morphology, an irregular border, and the loss of the fatty hilum (Figure 2). Size criteria for lymph node enlargement differ depending upon location. In general, lymph nodes with a short-axis diameter greater than 10 mm are considered suspicious for metastasis; however, in the inguinal region, lymph nodes measuring up to 15 mm can be considered normal, whereas lymph nodes exceeding 8 mm in the obturator region would raise suspicion [26]. Potential pitfalls arise in patients with increased body habitus; limited studies have demonstrated a correlation between normal lymph node size and body mass index (BMI), which can sometimes result in the overcalling of lymph node involvement [27].

According to the latest ESUR recommendations, T2WI and DWI sequences, ideally matched in acquisition plane, field of view, and slice thickness to allow for side-by-side interpretation, are fundamental for the initial staging, the assessment of treatment response, and the detection of recurrence. However, in the same guidelines, the use of contrast-enhanced MRI (CE-MRI) remains optional [15].

A systematic review by Avesani et al. did not find strong evidence to indicate contrast-enhanced (CE) sequences to be helpful in the initial staging or the detection of tumour recurrence and did not find CE-MRI could provide any additional information than that obtained from DWI sequences [28]. Combined chemoradiotherapy is the treatment of choice for large cervical cancers. MRIs are performed pre- and mid-treatment to enable tailored treatment planning and the adjustment of radiotherapy dose to improve local tumour control and minimize the toxic effect of therapy. If initial treatment fails, further therapeutic options are limited. Therefore, the early, accurate prediction of treatment response would profoundly affect the prognosis of patients. Many studies have investigated the potential role of CE-MRI at staging as a predictor of treatment response. Studies have shown that tumours demonstrating lower enhancement (poorly perfused hypoxic tumours are linked to increased aggressiveness, increased risk of metastasis, and treatment failure) had a poorer response to therapy and a lower survival rate. However, the studies did not identify a precise, reproducible value for those parameters, limiting their use in current clinical practice [28].

Some studies have also shown that CE-MRI can improve the sensitivity of depicting small isointense tumours, particularly for patients who may be eligible for fertility-sparing treatment [29]. A potential pitfall can arise in larger/exophytic tumours, where compression of the cervix/vagina can cause surrounding cervical oedema or inflammatory change that can be mistaken for parametrial invasion. 

Recently, there has been a growing interest in radiomics and its potential to add value to the discriminatory and prognostic evaluation of cervical cancer when using PET-CT and MRI. Radiomics refers to the technology that uses artificial intelligence and machine learning to extract large quantities of information from a series of medical images and convert them into calculated quantitative data. The extracted features can then be used as alternative markers for underlying gene expression patterns and tumour biological characteristics such as morphology and intra-tumour heterogeneity [30]. 

Several studies have investigated the use of MRI-based radiomics in cervical cancer with favourable preliminary results. Becker et al. reported that the textural parameter of the ADC map correlates with the differentiation of cervical cancer, which could then be used to predict survival [31]. A study by Wormald et al. found that radiomic features from ADC maps and T2WI could potentially predict recurrence in patients with stage I and II low-volume cancers [32]. Laliscia et al. found that radiomic features from T2WI are useful in predicting the prognosis of locally advanced cervical cancers [33]. Meta-analyses have also been carried out and support the value of MRI-based radiomics models in predicting lymph node metastases and lymph-vascular space invasion status in patients with cervical cancer pre-operatively [34].

More research is needed before radiomics is integrated into routine clinical practice. However, preliminary results are promising and demonstrate that MRI-based radiomic features can be useful in the preoperative prediction and prognosis of patients with cervical cancer.

## 3. FIGO STAGING with MRI

MRI has a limited role in the detection of cervical cancer and is usually only performed on patients with histological evidence of cervical cancer. Traditionally, the staging system was largely clinically and surgically based. However, the most recent update in 2018 has formally incorporated imaging as part of the criteria, giving added importance to MRI as a way of accurately measuring tumours, which has direct implications on the FIGO stage. MRI has a reported accuracy of 93% compared to 60% with clinical evaluation for accurate tumour measurement, and measurements should be given in three planes: craniocaudal (CC), antero-posterior (AP), and transverse (TS) [35]. MRI can also accurately identify the presence of parametrial and vaginal invasion, nodal involvement, and bladder and bowel invasion [36] (Table 2). 

## 4. FIGO Stage I

**Figure 3:**
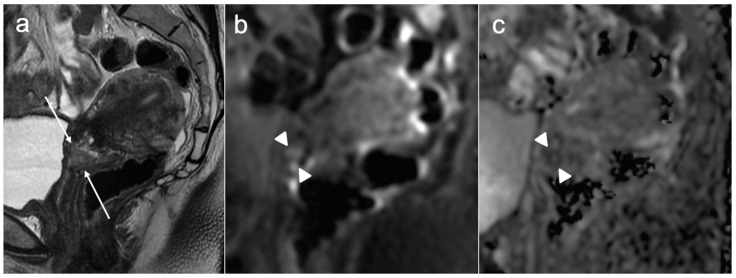
Sagittal T2WI (**a**) demonstrates a 15 mm intermediate signal intensity lesion in the anterior lip of the cervix (arrows). Corresponding DWI (**b**) and ADC map (**c**) show associated restricted diffusion (arrowheads). FIGO stage IB1.

**Figure 4:**
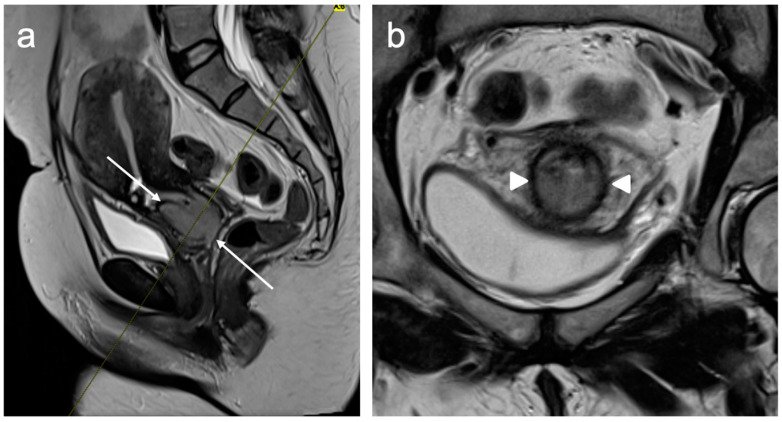
Sagittal T2WI (**a**) shows a well-defined intermediate signal intensity endocervical tumour (arrows); the maximum dimension is 28 mm. (**b**) Axial oblique T2WI through the mass demonstrates an intact low signal intensity stromal ring (arrowheads). FIGO stage IB2.

**Figure 5:**
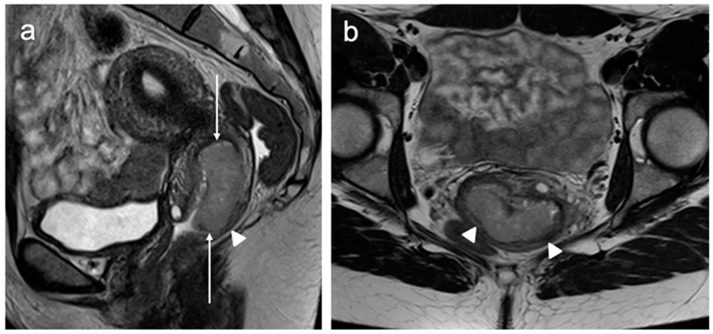
Sagittal T2WI (**a**) demonstrates a large 45 mm intermediate signal intensity lesion confined to the posterior lip of the cervix (arrows). Axial oblique T2WI (**b**) dearly reveals that the posterior vaginal wall is not involved (arrowheads). FIGO stage IB3.

A tumour confined to the cervix is considered stage I. Stage IA is a microinvasive disease that is not visible radiologically. Stage IB disease is a tumour that is confined to the cervix with the deepest invasion greater than 5 mm, and in the revised FIGO staging 2018, it is further divided into three subsections: stage IB1 disease is now defined as less than or equal to 2 cm in maximum diameter (Figure 3), stage IB2 is greater than 2 cm and less than or equal to 4 cm (Figure 4), and stage IB3 is greater than 4 cm in maximum diameter (Figure 5). These new subsections reflect the proven better prognosis for tumours under 2 cm and will include those who may be suitable for fertility-sparing treatment. 

## 5. FIGO Stage II

A tumour that extends beyond the cervix but without extension to the lower third of the vagina or pelvic sidewall constitutes stage II disease. There has been no change in stage II between the 2009 and 2018 FIGO classifications. Stage II is further subdivided into IIA and IIB. The stage IIA disease confers involvement of the upper two-thirds of the vagina; this can be challenging to assess radiologically, and vaginal invasion can be overestimated at MRI, particularly at the vaginal fornices, which may be stretched by a bulky exophytic cervical tumour. Overall accuracy for vaginal invasion is reported to be in the range of 86–93% [37]. The vaginal mucosa is normally of high T2 signal intensity, and when there is a loss of this signal in continuity with the primary tumour, vaginal invasion can be reported with confidence. Some centres advocate the use of vaginal gel to improve the accuracy of the involvement of the vagina; however, due to the accurate assessment of the vagina at examination under anaesthesia (EUA), this is not essential [13,38]. Stage IIA is further subdivided depending on the maximum size of the tumour: stage IIA1 comprises tumours measuring less than or equal to 4 cm, and stage IIA2 comprises tumours greater than 4 cm. This distinction relates to prognostication, as tumours that exceed 4 cm are more likely to have nodal metastases and are therefore unlikely to be surgical candidates. 

**Figure 6:**
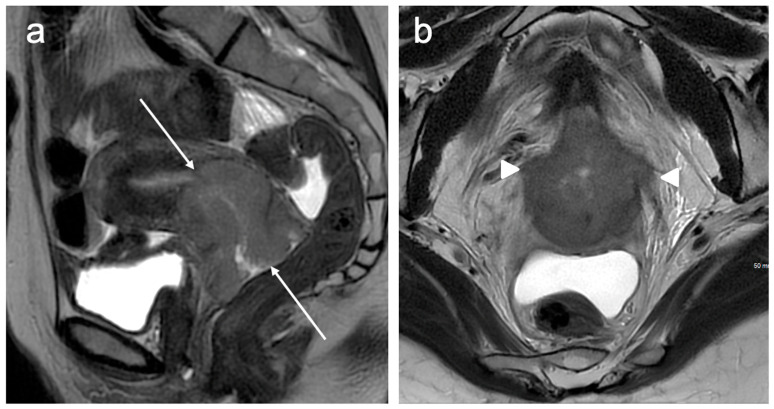
Sagittal T2WI (**a**) shows a large intermediate signal intensity tumour replacing the cervix and extending into the lower uterine segment (arrows). Axial oblique T2WI (**b**) reveals nodular soft tissue extension into the parametria bilaterally (arrowheads) consistent with bilateral parametrial invasion. FIGO stage IIB.

The parametrium is the fatty tissue containing blood vessels and lymphatics surrounding the cervix. Stage IIB disease constitutes parametrial invasion, but the tumour does not extend to the pelvic sidewall. The assessment of the parametrium is best depicted on axial oblique images, where normal cervical stroma is visualised as a low T2 signal ring, which, if intact, has a high negative predictive value (94–100%) for the presence of parametrial invasion [39]. However, when there is an isolated disruption of the stromal ring, parametrial invasion may not be present. The presence of stromal ring disruption and visible nodular or spiculate soft tissue extending into the parametrial soft tissues implies parametrial invasion (Figure 6). It is important to be aware of pitfalls, particularly with regard to post-biopsy cervical oedema, which can mimic parametrial invasion [22]. Whilst increasing the time interval between biopsy and MRI can reduce oedema, an unnecessary delay in imaging is not desirable. The use of DWI can overcome this challenge in distinguishing post-biopsy change from the tumour by identifying restricted diffusion within the tumour [40]. 

## 6. FIGO Stage III

**Figure 7:**
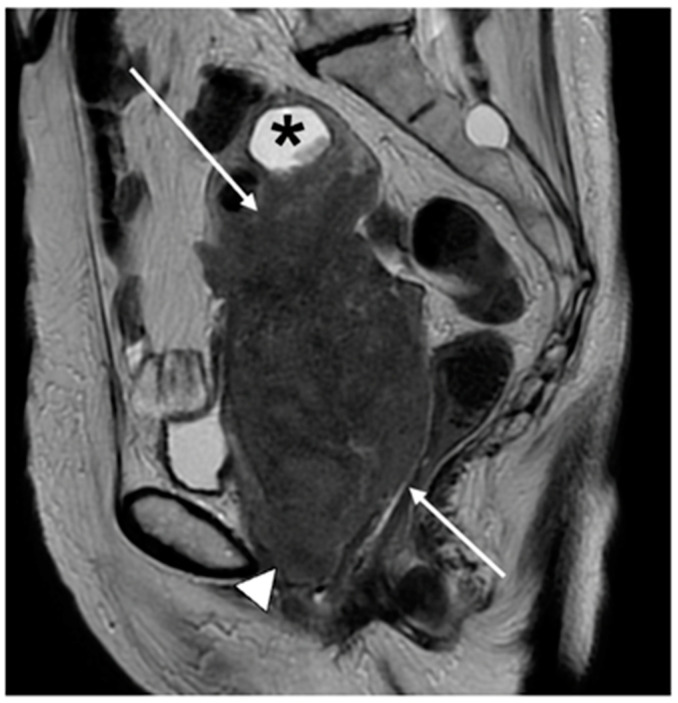
Sagittal T2WI of histologically confirmed cervical cancer demonstrates a large ill-defined intermediate signal intensity tumour replacing the cervix and the lower two thirds of the uterine body (arrows). Fluid distension of the fundal aspect of the endometrial cavity (*) secondary to cervical stenosis. Intermediate signal intensity also extends to and involves the lower third of the vagina (arrowhead). FIGO stage IIIA.

**Figure 8:**
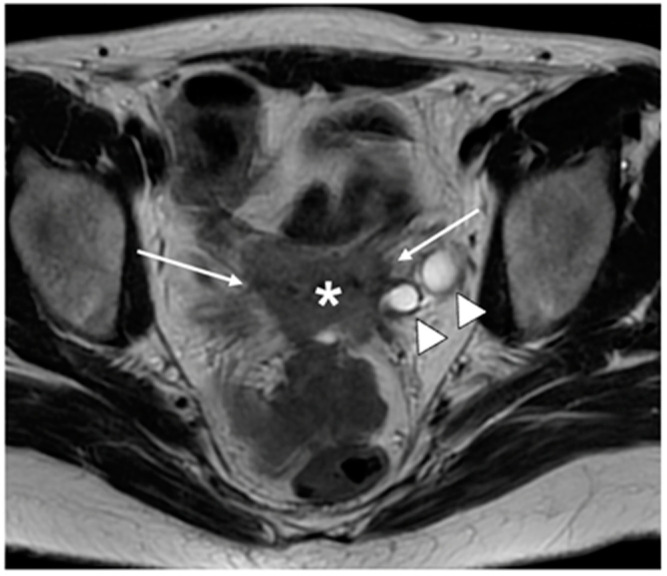
Axial T2WI shows an ill-defined intermediate signal intensity tumour replacing the cervix (*). Spiculated tumour extends into the parametria bilaterally (arrows) and causes a left hydroureter (arrowheads). FIGO stage IIIB.

**Figure 9:**
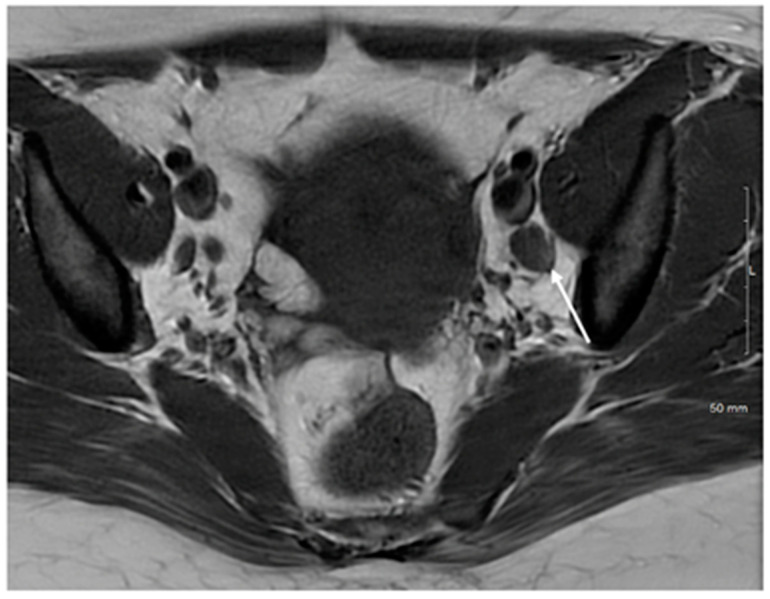
Axial T1WI demonstrating an enlarged left obturator node (arrow). FIGO stage IIIC1.

**Figure 10:**
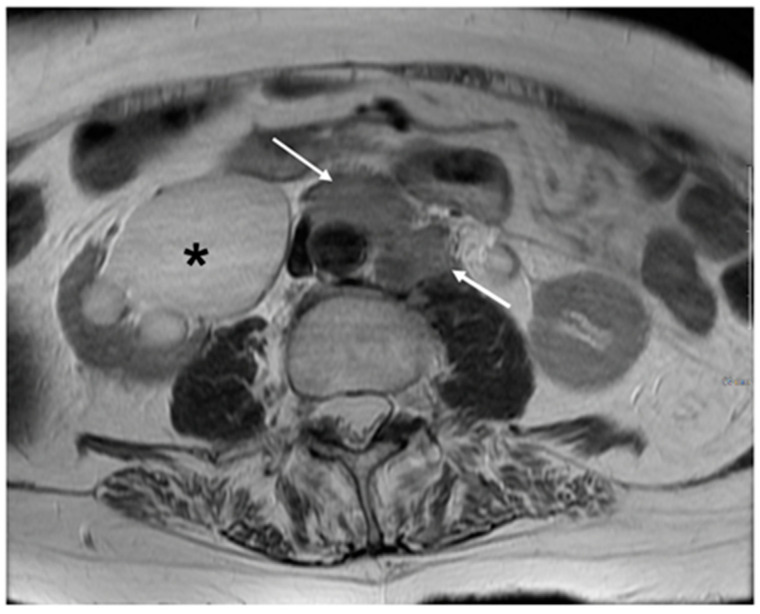
Axial T1WI demonstrating enlarged left para¬aortic and pre-aortic nodes (arrows). FIGO stage IIIC2. Right sided hydronephrosis also noted (*).

Stage III disease denotes further extension of the tumour and has three subsections. Stage IIIA represents an extension to the lower third of the vagina, which is the vaginal tissue below the level of the bladder base, best evaluated on sagittal T2WI or DWI (Figure 7). Stage IIIB is the extension of the tumour to the pelvic sidewall. The pelvic sidewall is bordered by the obturator internus and piriformis muscles and contains the iliac vessels, pelvic ureters, and lateral lymph nodes [41]. On MRI, a tumour within 3 mm of the lateral pelvic wall is considered a sidewall invasion. Stage IIIB also includes the presence of hydronephrosis or a non-functioning kidney (Figure 8); however, it is important to exclude other causes of hydronephrosis, such as endometriosis or urinary tract calculi, to avoid incorrect upstaging. Stage IIIC is a new substage and describes the pattern of abdominopelvic lymph involvement, regardless of primary tumour size and extent. It is further subdivided into IIIC1 (pelvic lymph node involvement, Figure 9) and IIIC2 (para-aortic lymph node involvement, Figure 10). The inclusion of nodal disease relates to prognostication, as patients with lymph node involvement have a significantly reduced 5-year survival rate compared to those without. Wright et al. reported that the five-year survival rate for stage IB1 tumours was accurate for 92% of patients, reducing to 61% for stage IIIC1 and to 38% for stage IIIC2 tumours [42].

## 7. FIGO Stage IV

**Figure 11:**
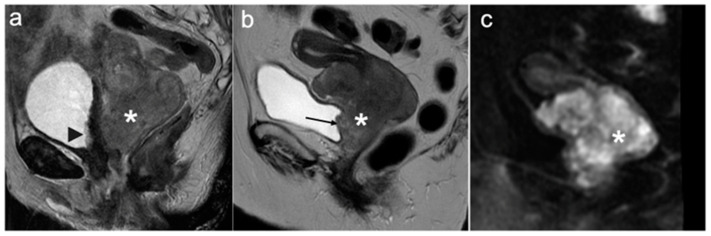
Sagittal T2WI (**a**) showing a heterogeneous intermediate signal intensity bulky cervical tumour (*). Irregular high T2 signal intensity seen along the posterior bladder wall consistent with bullous oedema (arrowhead). No tumour signal intensity is seen protruding into the bladder. Sagittal T2WI (**b**) with corresponding DWI (**c**) of a different patient demonstrating intermediate signal intensity bulky cervical tumour with corresponding diffusion restriction (*). Intermediate tumour signal intensity is seen to disrupt the low signal intensity of the posterior bladder wall and protrudes through the posterior bladder mucosa into the lumen (arrow) consistent with bladder invasion. FIGO stage IVA.

Stage IV disease is unchanged from 2009 and describes the disease extending into the adjacent organs outside the true pelvis. It is subdivided into two stages: stage IVA describes an extension of the tumour through the bladder wall anteriorly or rectum posteriorly. The tumour must be visualised to project beyond the mucosa into the lumen before describing stage IVA disease (Figure 11). MRI has a reported specificity of 86-88% for bladder/bowel involvement and a high negative predictive value of 96-100%, thereby removing the need for routine cystoscopy/sigmoidoscopy for staging [37]. If there is a loss of fat plane between the cervix and bladder or rectum or abnormal signal intensity of the serosa, this does not constitute stage IVA disease; however, this information should be conveyed to clinicians to prompt further evaluation, and cystoscopy or sigmoidoscopy in these cases would be beneficial. Bullous oedema, which describes the layered appearance of the posterior bladder secondary to urothelial oedema or inflammation, is a common pitfall and should not be mistaken for tumour infiltration. Bullous oedema is often seen in the presence of bladder serosal or muscularis invasion and is commonly seen post-radiotherapy (Figure 11).

**Figure 12:**
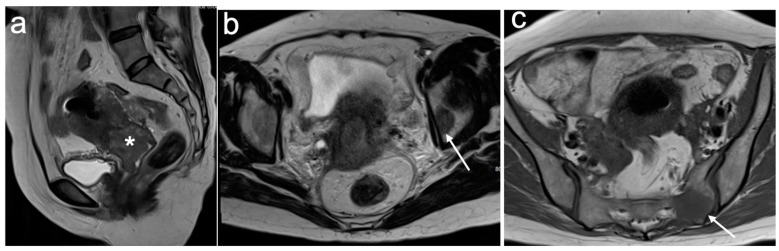
Sagittal T2WI (**a**) demonstrating intermediate signal intensity cervical tumour (*). Axial T2WI at the level of mid pelvis (**b**) and sacrum (**c**) demonstrates focal regions of irregular low signal intensity within the left acetabulum and left sacral ala (arrows) consistent with bone metastases. FIGO stage IVB.

Stage IVB describes metastases to distant organs (e.g., lung, bones, liver) or distant lymph node groups, such as those in the supraclavicular region (Figure 12). Importantly, inguinal nodes are also stage IVB disease, as these represent haematogenous spread.

The benefits of structured reporting have been shown to reduce inter-reader variability, reduce diagnostic errors, and improve communication with fellow clinicians [43]. We demonstrate a sample reporting template, incorporating the pertinent features for FIGO staging (Table 3).

## 8. Impact of MRI Findings on Treatment Selection

Cervical cancer is managed with curative intent, and the aim of FIGO staging is to risk stratify patients who are eligible for primary surgery and those who will have a better prognosis with chemo-radiation. Surgery is considered for patients where the tumour measures less than 4 cm and is confined to the cervix without parametrial or nodal invasion. For a select cohort of patients desiring the possibility of future pregnancy, fertility-sparing surgery may be an option. In such cases, tumour size, most accurately depicted with MRI, plays a significant role in determining which patients are eligible for fertility-sparing surgery. This is usually reserved for tumours confined to the cervix that measure less than 2 cm (stages IA1, IA2, and IB1) [44]. 

**Figure 13:**
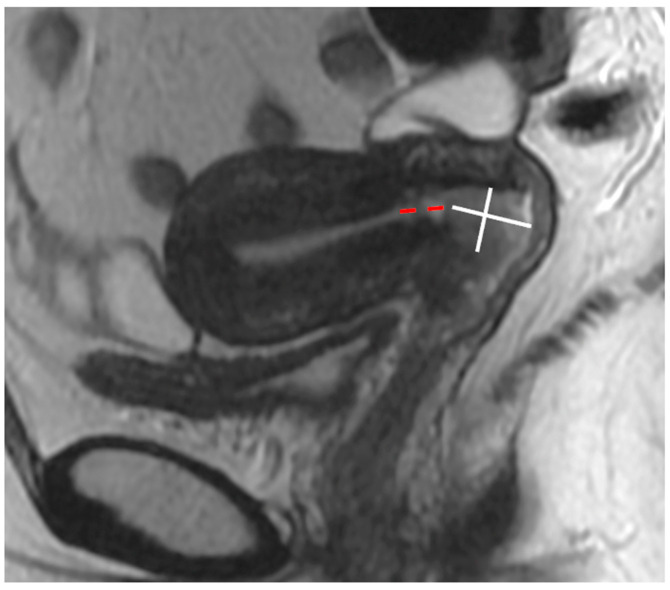
Sagittal T2WI demonstrating cervical tumour margins (white lines) and the distance from the internal cervical os and superior margin of the tumour (red dashed line).

Fertility-sparing surgery includes cone resection/cone biopsy, simple trachelectomy, or radical trachelectomy [39]. As well as tumour size, other criteria must also be met to be eligible: the distance between the cranial margin of the tumour and internal os should be greater than 1 cm; however, some centres accept a minimum distance of 0.5 cm [45]. MRI has a reported sensitivity of 91% and specificity of 97% in the evaluation of internal os involvement [46]. On sagittal T2WI, the internal os is seen as the narrowest point of the uterine body or the transition point where the low-signal intensity cervical stroma changes to the higher-signal intensity uterine myometrium. The distance between the superior margin of the tumour and the internal os is measured in the sagittal plane [47,48] (Figure 13). 

The depth of stromal invasion is also an important consideration and varies between centres, with some only accepting stromal invasion of less than 50% as part of their criteria [44]. 

Cone resection is the removal of the ectocervix and distal endocervical canal. It is usually performed for stage IA1 tumours without lymphovascular space invasion (LVSI). Simple trachelectomy involves the more extensive removal of the cervix. Radical trachelectomy involves excision of the cervix, vaginal cuff, and parametrium, followed by the creation of an anastomosis between the isthmus and vagina. Trachelectomy can be performed vaginally or abdominally via open or laparoscopic techniques and is the approach for stage IA1 tumours with LVSI, stage IA2, and stage IB1 tumours. 

For larger tumours confined to the cervix (exceeding 2 cm) or for patients for whom fertility preservation is not a priority, surgical options include total abdominal hysterectomy with or without bilateral salpingo-oophorectomy and lymphadenectomy via open or laparoscopic routes. Patients with FIGO stages IB2 and IIA1 may also be offered primary chemoradiotherapy if they are considered less favourable surgical candidates [3]. However, a systematic review by Yan et al. demonstrated radical hysterectomy to be superior to chemoradiotherapy for stage IB1, IB2, and IIA1 cancers with regards to overall prognosis [49] 

A tumour size of greater than 4 cm (IB3, IIA2), among other factors, increases the risk of lymph node metastases and parametrial invasion [20,21]. Whilst some patients may be considered for surgery, the risk of recurrence and thus the need for adjuvant radiotherapy is greater (which is associated with higher morbidity); therefore, primary chemoradiotherapy is often the preferred treatment option [3].

**Figure 14:**
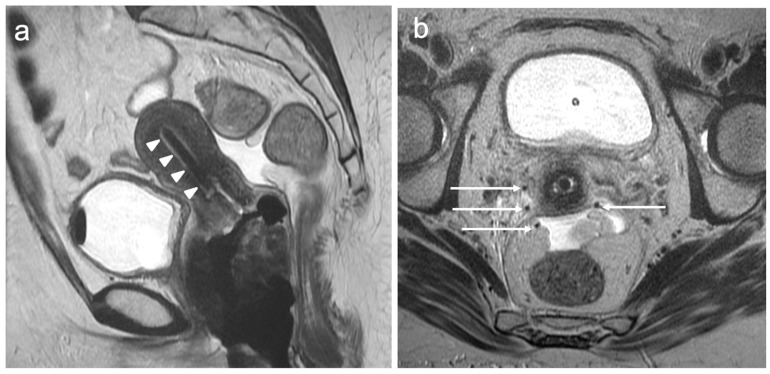
Sagittal T2WI (**a**) demonstrating the brachytherapy applicator appropriately sited within the endometrial cavity (arrowheads). Axial T2WI (**b**) shows several appropriately positioned parametrial needles (arrows).

**Figure 15:**
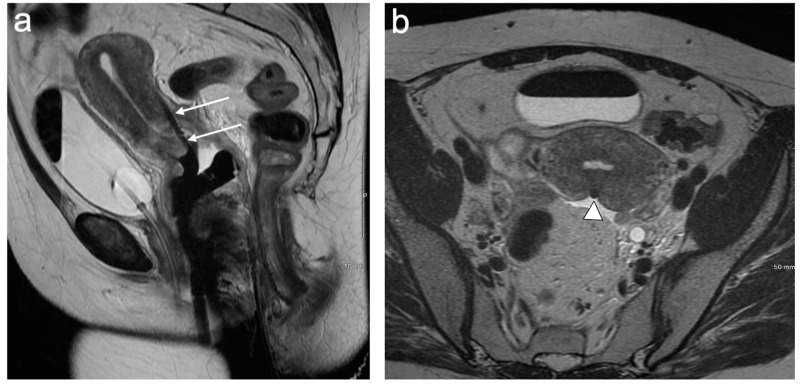
Sagittal T2WI (**a**) and axial T2WI (**b**) demonstrates a malpositioned central brachytherapy applicator which courses through the posterior cervical wall (arrows) with the tip lying posterior to the uterine body (arrowhead).

**Figure 16:**
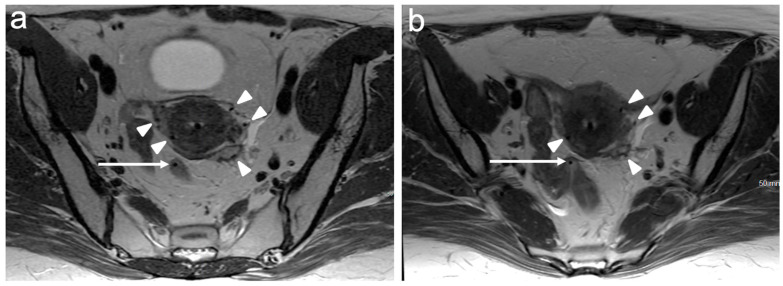
Axial (**a**) and oblique (**b**)T2WI demonstrates a single right sided parametrial needle to be malpositioned, perforating into the sigmoid colon (arrows). The remaining parametrial needles are appropriately sited (arrowheads).

**Figure 17:**
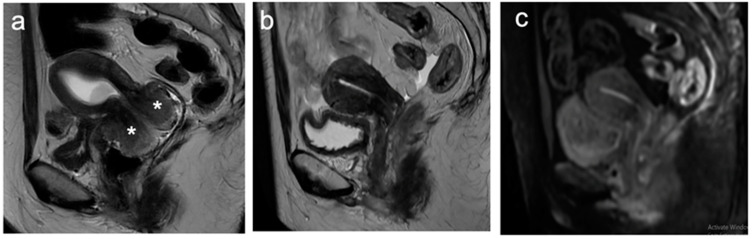
Baseline sagittal T2WI (**a**) shows an intermediate signal intensity bulky, exophytic cervical tumour (*). Sagittal T2WI (**b**) and DWI (**c**) after completion of chemoradiotherapy demonstrates significant reduction in tumour size with no residual abnormal signal intensity or diffusion restriction. This appearance indicates a complete response.

The standard treatment for locally advanced cervical cancer (stage IIB and above) is chemoradiotherapy, which involves external beam radiation (EBRT) with concurrent chemotherapy, followed by intracavitary brachytherapy. MRI can be used to assess for accurate placement of the brachytherapy applicators (Figure 14) and post-brachytherapy complications (Figure 15 and Figure 16). For patients treated with chemoradiotherapy, interval imaging can be used to monitor disease response (Figure 17). Mid-treatment MRI (after approximately 5 weeks of commencing chemotherapy with EBRT and before intracavitary brachytherapy) can aid dose adjustment in proportion to the residual tumour volume, which can reduce toxicity. Post-treatment MRI is typically performed 3–6 months after chemoradiotherapy, and the reconstitution of the low-signal intensity cervical stroma on T2WI implies a complete response. Post-treatment changes can persist for up to 9 months post-chemoradiotherapy; therefore, distinguishing residual tumours from post-treatment oedema can be challenging as both will appear as intermediate signal intensities on T2W1. The use of DWI can help differentiate the two, as only tumours should demonstrate restricted diffusion [50]. More recently, the application of MRI-guided brachytherapy has been shown to deliver more accurate dosing, which can be individually tailored to the tumour volume, thereby improving overall morbidity [51,52,53]. 

At initial staging, whole-body FDG-PET/CT is recommended for patients with stage IB3 and above due to the higher incidence of extra-pelvic disease. PET/CT allows accurate assessment of lymph node involvement and has a reported sensitivity and specificity of 72% and 96%, respectively [12]. PET/CT further optimises patients’ triage to the appropriate therapy [10]. For example, patients with para-aortic nodal involvement have been shown to have a survival benefit when treated with extended-field radiotherapy. For bulky lymph nodes, standard EBRT may be insufficient, and these patients may be offered high-dose boost irradiation as part of standard chemoradiation or nodal debulking to reduce the overall dose of radiation required [54]. 

## 9. Recurrent Cervical Cancer

**Figure 18:**
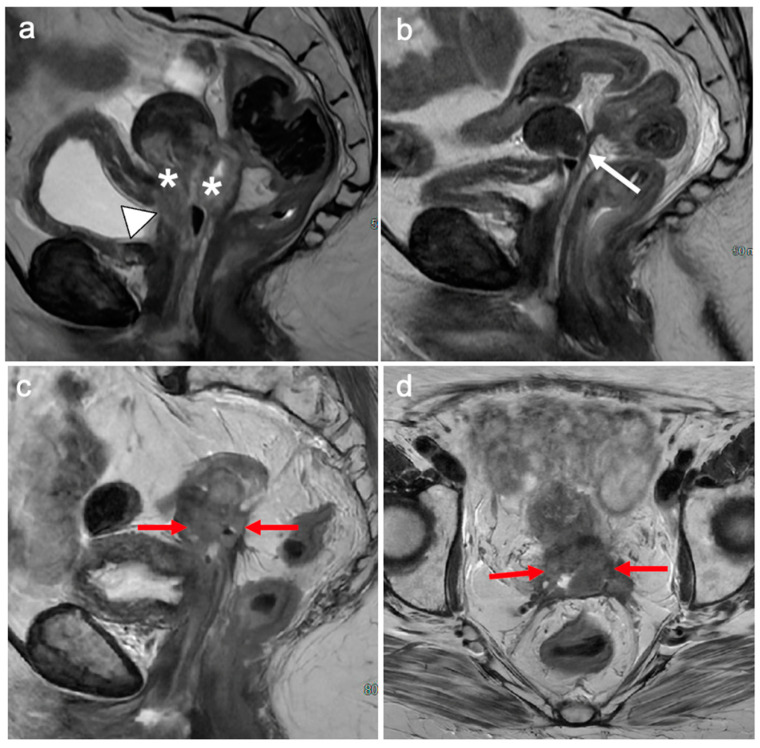
Sagittal T2WI at initial staging (**a**) demonstrates a bulky cervical tumour (*) with bladder involvement (arrow head). 6 month post-treatment (**b**) demonstrates reconstitution of the low signal cervical stroma indicating a complete response. Thin low signal intensity bands extend to the rectum consistent with post treatment fibrosis (white arrow). One year later the patient began experiencing bowel symptoms and subsequent MRI in sagittal (**c**) and axial (**d**) planes demonstrates intermediate signal intensity at the cervix (red arrows), extending to and involving the rectum consistent with recurrence.

Recurrent cervical cancer is defined as tumour regrowth or the development of nodal or distant metastases more than six months after the primary lesion has regressed or been resected [55]. Cervical cancer typically recurs early, and in 60–70% of cases, it recurs within 2 years of starting treatment [18]. Recurrence may be loco-regional, including recurrence in the vaginal vault, cervix, uterus, bladder, bowel, or involving pelvic nodal stations. Distant recurrence includes the involvement of extra pelvic nodal stations (e.g., paraaortic, supradiaphragmatic) or distant organ metastases. There is no established role for routine imaging follow-up for patients treated with hysterectomy. Imaging is usually reserved for patients with a clinical suspicion of recurrence, such as those who present with abnormal vaginal discharge or bleeding. The commonest sites of recurrence post-hysterectomy include the vaginal stump and rectovaginal space. After fertility-sparing surgery, patients are imaged with MRI at six months and then annually for 2–3 years. After chemoradiotherapy, patients are reimaged with FDG-PET/CT and MRI at 3–6 months [50]. The protocol is similar to that used in initial staging; however, the axial oblique planes are adjusted to the vaginal vault in the setting of prior hysterectomy. The recurrent tumour has a similar appearance to that of the primary tumour and appears as intermediate signal intensity lesions on T2WI with corresponding diffusion restriction on DWI and ADC maps (Figure 18). Radiotherapy-induced fibrosis usually demonstrates low signal intensity on all sequences. However, in some cases, the signal intensity may be atypical and therefore it can be difficult to differentiate fibrosis from tumour. DWI is helpful in this scenario, as fibrosis does not demonstrate restricted diffusion. Post-contrast imaging is less useful as both fibrosis and tumour can demonstrate enhancement.

## 10. Conclusions

MRI is integral to the management of cervical cancer, having been formally incorporated into the updated 2018 FIGO staging system. MRI allows the accurate assessment of tumour size, parametrial involvement, and lymph node involvement, which are crucial for triaging patients into those that will be eligible for primary surgery or chemoradiotherapy. MRI has applications for radiotherapy planning and image-guided adaptive brachytherapy. It also has a role in evaluating treatment response and detecting tumour recurrence and possible treatment complications.

## Figures and Tables

**Table 1:** Recommended MRI sequences for staging of cervical cancer.

Sequence and Plane	Rationale
Large FOV Axial T1WI	Extra pelvic disease, lymph nodes, bone marrow signal
Large FOV Axial T2WI	Extra pelvic disease, para-aortic nodal involvement, hydronephrosis
Small FOV Sagittal T2WI	Accurate tumour size, local staging (e.g., vaginal, bladder, rectal invasion)
Small FOV Axial oblique T2WI	Local staging, parametrial and pelvic sidewall involvement
Sagittal and axial oblique DWI and ADC maps (corresponding to sagittal and axial oblique T2WI)	Identifying small isointense tumours, unsuspected bone metastases

**Table 2:** FIGO staging 2018.

Stage	Description
Stage I	The carcinoma is strictly confined to the cervix
IA	Invasive carcinoma that can be diagnosed only by microscopy with a maximum depth of invasion <5 mm
IA1	Measured stromal invasion <3 mm in depth
IA2	Measured stromal invasion ≥3 mm and <5 mm in depth
IB	Invasive carcinoma confined to the uterine cervix with measured deepest invasion ≥5 mm
IB1	Tumour measures <2 cm in greatest dimension
IB2	Tumour measures ≥2 cm and <4 cm in greatest dimension
IB3	Tumour measures ≥4 cm in greatest dimension
Stage II	The cervical carcinoma invades beyond the uterus, but has not extended onto the lower third of the vagina or to the pelvic wall
IIA	Involvement limited to the upper two-thirds of the vagina without parametrial invasion
IIA1	Invasive carcinoma <4 cm in greatest dimension
IIA2	Invasive carcinoma ≥4 cm in greatest dimension
IIB	With parametrial invasion but not up to the pelvic wall
Stage III	Involves the lower third of the vagina and/or extends to the pelvic wall and/or causes hydronephrosis or non-functioning kidney and/or involves pelvic and/or paraaortic lymph nodes
IIIA	Involves lower third of the vagina, with no extension to the pelvic wall
IIIB	Extension to the pelvic wall and/or hydronephrosis or non-functioning kidney (unless known to be due to another cause)
IIIC	Involvement of pelvic and/or paraaortic lymph nodes
IIIC1	Pelvic lymph node metastasis only
IIIC2	Paraaortic lymph node metastasis
Stage IV	Spread to adjacent and distant organs
IVA	Rectal or bladder involvement
IVB	Spread to distant organs outside the pelvis

**Table 3:** Sample structured report for cervical cancer staging.

MR Cervical Cancer Staging	
Uterus size	CC × AP × TS mm
Primary tumour	Not seen (0), Ectocervical (exophytic 1), Endocervical (endophytic 2), Infiltrative (1 predominant expansive or 2 predominant infiltrating)
Size	CC × AP × TS mm
Presence of necrosis	No Yes (diameter)
Parametrial invasion	No Yes: Left/Right/Bilateral (proximal or distal)
Uterine invasion	No Yes: Lower/Mid/Upper
Extension to vagina	No Yes: Upper 1/3/Mid 1/3/Lower 1/3
Hydronephrosis	No Yes: Left/Right/Bilateral
Pelvic sidewall invasion	No Yes: Left/Right/Bilateral
Bladder invasion	No Yes
Rectal invasion	No Yes: Mesorectum/rectal wall
Distant organ invasion	No Yes
Lymph nodes	None, External Iliac, Internal Iliac, Obturator, Inguinal, Para-Aortic (above/below renal hilum)
Additional findings	
FIGO STAGE 2018	IA IB1 IB2 IB3 IIA1 IIA2 IIB IIIA IIIB IIIC IVA IVB

## Data Availability

No new data were created or analyzed in this study. Data sharing is not applicable to this article.
